# The Fungal, Nutritional, and Metabolomic Diagnostics of the Oil Palm *Elaeis guineensis* Affected by Bud Rot Disease in Esmeraldas, Ecuador

**DOI:** 10.3390/jof9090952

**Published:** 2023-09-21

**Authors:** Raluca A. Mihai, Erly J. Melo Heras, Pablo A. Landazuri Abarca, Rodica D. Catana

**Affiliations:** 1 CICTE, Department of Life Science and Agriculture, Universidad De Las Fuerzas Armadas—ESPE, Av. General Rumiñahui s/n y Ambato, Sangolquí 171103, Ecuador; 2 Department of Life Science and Agriculture, Universidad De Las Fuerzas Armadas—ESPE, Av. General Rumiñahui s/n y, Sangolquí 171103, Ecuador; ejmelo@espe.edu.ec (E.J.M.H.);; 3 Institute of Biology Bucharest, Romanian Academy, 060031 Bucharest, Romania

**Keywords:** fungi, bud rot, nutrition status, palm oil, 16S, metabolomics, DRIS, HPLC-MS

## Abstract

The oil palm *Elaeis guineensis* represents one of the most important crops in Ecuador. Considering that bud rot disease is deadly in Ecuador, more attention has been given to identifying possible causes for palm debility from this disease. We studied the involvement of fungi and nutrients in triggering bud rot disease in *E. guineensis.* Special emphasis was given to the molecules synthesized by the plant to protect against this devastating disease. Techniques like Diagnosis and Recommendation Integrated System (DRIS) and metagenomic analysis were used to understand the possible implications of biotic and abiotic factors in the development of bud rot disease in oil palm in Ecuador. Liquid chromatography-mass spectrometry (LC-MS) analysis was used to identify the phenolic protection barrier of the palm facing the disease. Our results indicate that fungi from *Ascomyceta* phylum were found in the tested samples. The species directly involved are different in soil compared with plants. The results indicate a deficiency of chemical elements, such as Ca, Mn, Mg, and Fe, which are responsible for palm debility from bud rot disease. More than 30 compounds with protective roles were identified in the leaves of symptomatic plants from the first stage of the infection.

## 1. Introduction

*Elaeis guineensis*, the African oil palm native to West Africa, is the most important palm species, being the world’s highest-yielding edible oil crop used in the food and nonfood sectors [1]. The interest in *E. guineensis* is given by its economic importance because it is the world’s largest edible oil [2] with numerous medicinal values [3].

At the global level, palm oil crops are dealing with numerous pressures [4], like pathogens represented by fungi, bacteria, viroids, and viruses [5], which are affecting the oil palm by reducing yield or retarding growth [6]. At various times, oil palm plantations from southeast Asia, Africa, and South America were affected by almost 32 diseases and disorders [7]. Among diseases, bud rot is a catastrophic one affecting more than 50% of the plantations [8]. Bud rot is one of the two important phytosanitary problems in tropical America. 

But rot destroys the young tissue of the plants; the lesions appear to descend from the middle of unopened internal leaves to the meristematic zone followed by chlorosis of the youngest leaves, necrosis, and plant death [9]. Once the lesions progress, colonization with pathogens occurs [10]. Until now, two forms of bud rot disease have been found: a lethal one (in Ecuador, Brazil, Colombia, and Suriname) and a nonlethal form with a good recovery rate (in Colombian Llanos) [11]. The highest incidence in Ecuador is in the coastal areas, especially in the province of Esmeraldas, where the temperature and relative humidity are naturally favorable for this disease’s high incidence and spread. Starting in the 2020s, bud rot has been the main cause of deterioration and loss of plantations in Ecuador.

In 2020, Ecuador was the 17th largest producer worldwide of oil palm and the 15th largest exporter in the world [12] due to its growing and stable culture. Ecuador has 200,908 hectares (496,454 acres) of oil palm plantations, 40% in Esmeraldas, 18.5% in Los Ríos, and 10% in Santo Domingo. Esmeraldas is characterized by the highest rate of deforestation in the country and also by the largest oil palm plantations in Ecuador according to the National Institute of Statistics and Census, 2019 [13]. 

Even if bud rot symptoms are easy to recognize, diagnostic confirmation is difficult due to the pathogen’s colonization, which makes it difficult to identify the agents and therefore the possibility of applying a treatment [9]. Two types of factors were identified for this disease: biotic and abiotic factors. At the international level, bacteria *(Erwinia* spp.) [14], fungi (*Thielaviopsis* spp. and *Fusarium* spp.) [15], and Oomycetes *(Pythium* spp. and *Phytophthora* spp.) [10] were identified as being the organisms involved in bud rot disease. The nutrition status may represent the abiotic factor [16,17]. An improper level of minerals might trigger susceptibility to beneficial microorganisms. In this context, there is a lack of studies on the nutritional factors affecting bud rot disease in Ecuador. 

Successful disease management consists in the use of resistant planting material [18], proper drainage, good fertilization, intense monitoring, and rapid agronomic interventions (destruction of infected palms) [19]. Before establishing a new plantation, farmers are advised to improve their drainage systems and to analyze the soil’s biodiversity [20]. 

An important factor in plant development is played by the rhizosphere microbiome [21]. The studies concerning the association between *E. guineensis* and microbial communities were focused on the use of bacteria isolated from the rhizosphere to promote plant growth or control the white rot fungus *Ganoderma boninense* [22].

MiSeq sequencing is a molecular technique that detects and identifies fungal and bacterial species from different samples, like plants, food, water, and soil [23].

Secondary metabolites represent the molecules identified in different aspects of the plant. One of the major roles of these molecules is plant protection against different types of stress, such as biotic (bacteria, fungi, nematodes, and insects) and abiotic (temperature, moisture, shading, heavy metals, and different levels of nutrients) [24].

This investigation is the first one that tries to demonstrate the biotic (fungi) and abiotic (nutritional involvement) factors involved in bud rot disease development in the oil palm plantations from the Esmeraldas area. Also, important aspects concerning the secondary metabolites in the symptomatic plants were investigated. 

## 2. Materials and Methods

### 2.1. Study Area

Ecuador has 8149 oil palm plantations, and the province of Esmeraldas is the one with the highest production with 3280 plantations and 116,430.38 hectares, while Quinindé Canton represents 37% of plantations at the national level and 91% at the provincial level [25]. The Canton is located 100 km from the Province of Esmeraldas, to the southeast of its territory, at 00°13′33″ N Latitude, 73°26′00″ W Longitude, and it has an average height of 115 m.a.s.l., a 21–31 °C temperature range, and an average annual precipitation of 2300 mm, except for abnormal periods, such as the El Niño phenomenon [26]. The environmental climatic conditions are represented by relatively mild temperatures in winter, a warm summer, and rainfall distributed throughout the year. The dominant coverage of the study area is 52.2% forest and 44.8% agriculture [27].

The soil in the study area (78% medium-high fertility soil and 22% low-fertility soil) is represented by sediments from the ancient plains and mountain ranges of the coastal region, with a greater amount of water per volume of soil, greater retention of ions in interchangeable form, and greater resistance to leaching processes [28].

### 2.2. Foliar Sample Collection

The plant material was represented by the middle part of leaf number 17 of the oil palm *Elaeis guineensis* Jacq. adult plants collected from three plantations, in Ecuador, in July 2022, a period of low rainfall, and no fertilization, which may reduce the variability of the results. The harvested leaflets were cleaned before drying (at 70 °C), ground in a stainless-steel Wiley mill, and analyzed for total N by micro-Kjeldahl. An atomic absorption spectrophotometry was used for K, Ca, Mg, Fe, Zn, and Cu. A colorimetric method using vanadate molybdate reaction was used to detect phosphorus, a turbidimetric method for boron, and a photometry method for sulfur. Our results expressed in % (macronutrients) and mg/kg (micronutrients) were compared with the standards developed by Marrocos et al. (2020) [29].

### 2.3. Soil Sample Collection

The soil samples were collected in the morning hours, from the same palm sample used for the foliar analysis, consisting of 10 cores to a depth of 30 cm, and transported in plastic bags disposed of in cooler recipients to the laboratory for further analysis of pH, phosphorus (P), calcium (K), calcium (Ca), and magnesium (Mg) using the standard procedures of the Belle Glade, AREC—Agricultural Research and Education Center. For the determination of soil pH used in a 1:2 soil–water suspension, an AI block with nitric and perchloric acids was used to digest the ground sample [30]. Total P was determined by the molybdovanadophosphate colorimetric procedure [31] and K, Ca, Mg, Fe, Mn, Zn, and Cu were determined by atomic absorption spectrophotometry. 

### 2.4. DRIS Analysis

The DRIS (Diagnosis and Recommendation Integrated System) method uses “nutrient ratios” instead of absolute or individual nutrient concentrations for the interpretation of tissue analysis. The use of leaf nutritional assessment based on DRIS, in addition to the traditional methods such as the levels of critical ranges, is an important tool that mitigates the distortions of diagnoses caused by the effect of dilution, concentration, age, or plant organ [32]. To carry out the DRIS, an average of the foliar analyses was calculated to have a global reference for the area under study. Once the DRIS indices were obtained, the IBNM (analysis of nocturnal basal impedance) was obtained based on the formula:

(1) $$IBNa=\frac{IA+IB+IC+\cdots IN}{Z}$$

where IBN—the nutritional balance index, I—index, a—average, and Z—the number of indexes analyzed.

The IBNa with the standard deviation of the indices allows us to obtain the Potential Responses to the Application of Nutrients (RAPNs) [33]. The methodology used for the Potential Response to the Application of Nutrients was described by De La Torre, 2012 [33], where the absolute value of Ix was taken and transformed to ln, since IBMa and Ix are distributed exponentially according to their dry mass. RAPNs were obtained by subtracting the Ln/lx from Ln/IBMa| for each of the indices and to obtain the limits, SD|IBNa| (standard deviation) the upper and lower limits according to this definite integral (Table 1). According to Beaufils, 1973 [34], the sum of the DRIS indices is constrained to zero.

(2) $$\int_{\alpha }^{\beta }\frac{1}{\sigma \sqrt{2\pi }}e_{\;\;\;\;\;\;\;\;\;\;\;2a2}^{\left[\left(Ix\right)i-IBNa\right]2}dx$$

### 2.5. Metagenomic Analysis

Samples used for metagenomics were represented by soil (S1 and S2) and leaves (S3 and S4) collected from an *E. guineensis* plantation older than 4 years old. The soil samples were collected around the symptomatic plants (in stage 1) (S1) and healthy plants (S2). The plant samples were represented by symptomatic plants (in stage 1) (S3) and healthy plants (S4). The analysis was performed by BioSequence Ecuador. 

For the extraction of fungal genome DNA, leaves were collected after the previous identification of the disease stage, placed in aseptic bags, and kept at low temperatures to prevent senescence while being transported to the laboratory. Leaf samples from each plant were disinfected by applying a series of washing steps, as mentioned by Badotti et al., 2017 [35], which consisted of 70% (*v*/*v*) ethanol for 1 min, 3% (*v*/*v*) sodium hypochlorite solution for 3 min, 2.5% (*w*/*v*) sodium thiosulfate for 5 min, and rinsing the samples five times with sterile water. 

#### 2.5.1. DNA Extraction 

For DNA extraction, leaf samples were ground into a fine powder by using liquid nitrogen in a sterilized mortar and transferred into a bead tube for total DNA extraction. For subsequent analysis, DNA was stored at −20 °C. Target-specific primers were chosen from the MiSeq Illumina platform according to recommendations for fungal metabarcoding (Table 2), using indexes from the Nextera XT Index Kit v2 (Illumina, Catalog No. FC-131-2001).

The PCR amplification was performed according to Siddique et al., 2022 [36] for a 25 µL PCR mixture, which consisted of 1X Dream Taq buffer, 0.16 µM dNTP mix, 0.4 µM forward and reverses primers, 0.25 µg template DNA and 0.75 unit Dream Taq DNA polymerase and PCR grade water. The PCR reaction was conducted as described by Al-Bulushi et al., 2017 [37] with the next settings: an initial denaturation step of 95 °C for 5 min, 25 cycles of denaturation at 94 °C for 30 s followed by an annealing step at 54 °C for 40 s and extension step at 72 °C for 1 min, final extension 10 min at 72 °C.

#### 2.5.2. MiSeq Illumina Sequencing

For the sequencing, the method described by Hoggard et al., 2018 [38] was followed. A purification step was conducted for the initial PCR reaction using an Axygen PCR cleanup kit (Axygen), and then the quality was verified with 1% Thermo Fischer Scientific Massachusetts, U.S. agarose gel electrophoresis. The purified solution was diluted; then, it was used in a range of 50 to 100-fold as a new template for a second PCR under similar conditions to the first PCR, except for using 10 cycles as recommended by Al-Sadi and Kazerooni, 2018 [39]. For this PCR round, the Illumina Nextera PCR primers described in Table 3 were used, which were followed by quantification with a Quantus^®^ by Promega (Promega, Quito, Ecuador)). Amplicons were pooled and submitted for sequencing using an Illumina MiSeq (Illumina, Inc., San Diego, CA, USA).

### 2.6. Bioinformatics and Data Analysis

Adapters can pose a problem for library preparation on Illumina (FASTQ), so Trimmomatic is used to trim Illumina data (FASTQ) and remove adapters. The PAIRED END mode will keep read pairs matched and will also use the additional information contained in the paired reads to better find the adapter or PCR primer fragments introduced by the library preparation process [40]. FLASH (Galaxy Version 1.2.11.4) software was used to splice the reads of each sample; then, data were processed with the Trimmomatic (Galaxy Version 0.38.1) software to filter the spliced raw tags to obtain high-quality tags as described by Fan et al., 2020 [41]. Subsequently, identification at the species level was conducted through BLAST+ after downloading the UNITE database v8.2 [42,43].

### 2.7. LC-MS

#### 2.7.1. Extraction Process

Plant material (leaves from symptomatic plants—in stage 1) was dried by lyophilization at −57 °C and 0.50 hPa for 48 h before extraction and stored at 4 °C in plastic tubes. Dried and ground solid residue samples (1 g) were extracted with 20 mL of 80% methanol for 2 h at 30 °C as recommended by Irakli et al. (2021) [44]. The extract was centrifuged at 5000 rpm for 10 min at 4 °C (Eppendorf 5490 centrifuge, Hamburg, Germany); then, it was filtered and concentrated with a rotary evaporator (Buchi, New Castle, DE, USA). The concentrated extract was stored at −20 °C until analysis.

#### 2.7.2. LC-MS Analysis 

Dry filtrates were diluted to 1000 mg/L and filtered with a 0.47 µm microfiber filter before LC-MS analysis. The injection volume was 5 µL through an Accucore Vanquish 150 × 2.1 mm column. The mobile phase consisted of 0.1% formic acid in water (*v*/*v*) (Solvent A) and acetonitrile (Solvent B) with a mobile phase flow rate of 0.5 mL/min, as described by Kang et al. (2016) [45]. The phenolic compounds were identified with accurate retention time, according to the mobile phases used by Bikoro Bi Athomo et al., 2021 [46].

## 3. Results

### 3.1. DRIS Analysis Based on the Soil and Foliar Determinations 

**Figure 1:**
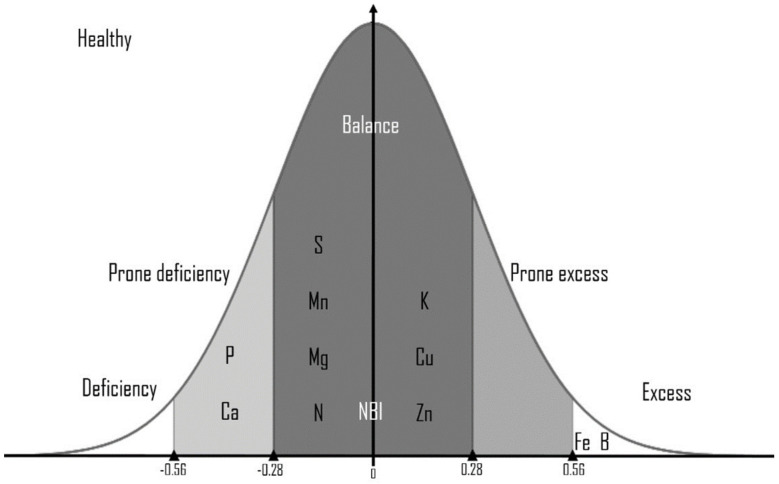

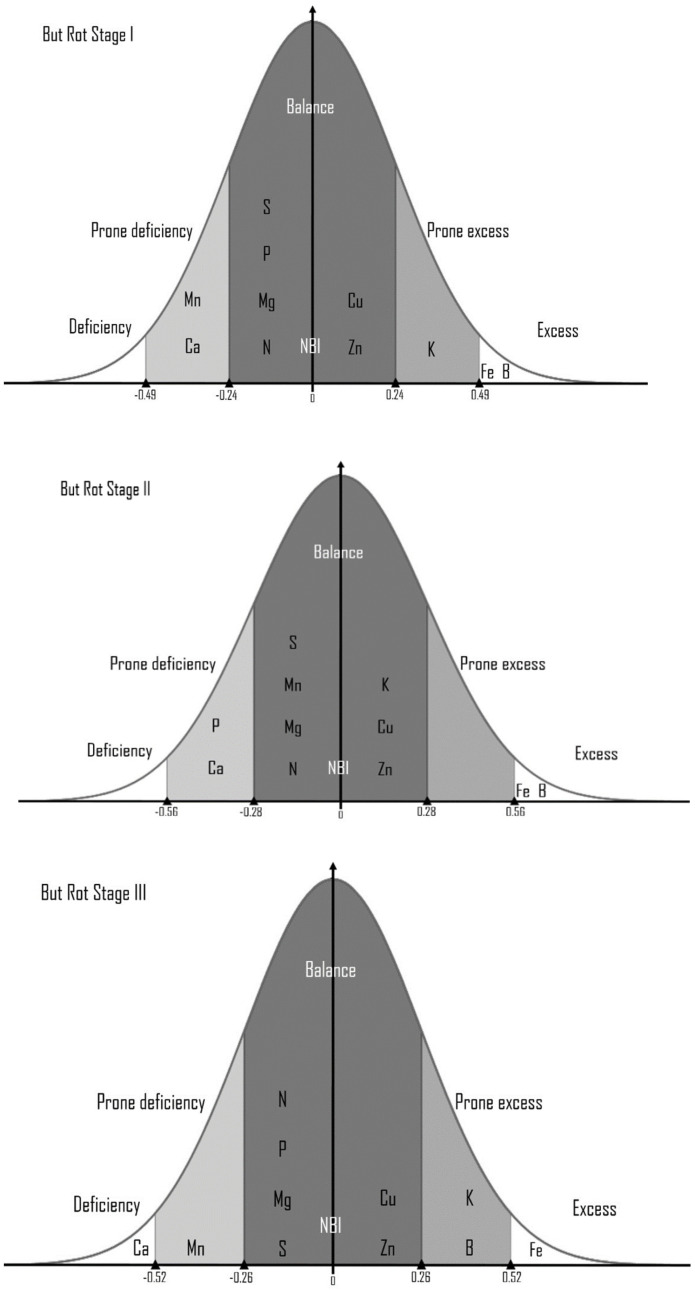
The soil nutrient variation on *E. guineensis* faces different bud rot disease stages.

Table 4 and Figure 1 present the DRIS indexes of nutrients to serve as a guide to quickly diagnose the nutrients needed by the palm which can influence its response in front of pathogens, making it resistant or feeble.

The nutritional balance index found in our samples showed a deficiency of natrium, potassium, calcium, magnesium, sulfur, and mangan in the case of all samples of healthy and symptomatic plants in all three stages of the bud rot. In stage II, leaves showed a higher value of deficiency in natrium (−517.24), calcium (−360.95), and mangan (−872.40), while leaves in stage III showed low levels of magnesium (−411.53) and sulfur (−430.52). The healthy plants showed a higher value of the indices of phosphorus (−622.36) (Table 4).

### 3.2. Metagenomic Analysis 

**Figure 2:**
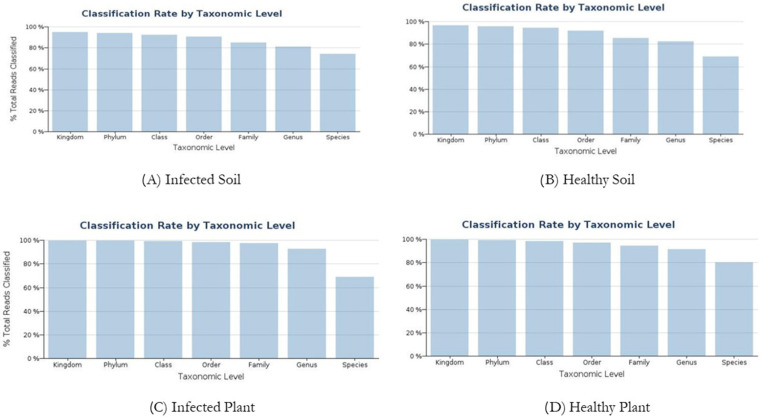
Histogram describes the number of species and sequences distributed per kingdom, phylum, class, order, family, genus, and species.

MiSeq outcome for the ribosomal ITS1 region was analyzed for each sample, obtaining 13.591 reads for the infected soil sample (A), 20.733 reads for the healthy soil sample (B), 292.437 reads for the infected plants sample (C) and 257.989 reads for the healthy plants sample (D). Each group per sample consists of 100% of Reads Passing Quality Filtering (Figure 2).

**Figure 3:**
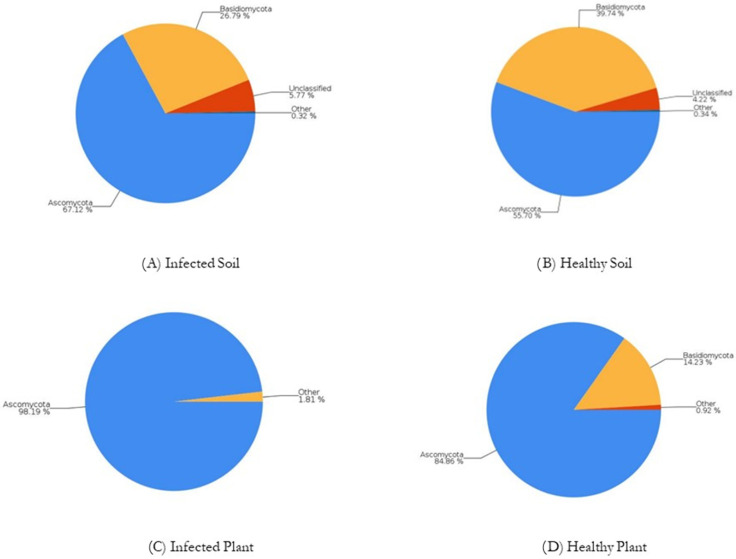
The abundance (%) of sequences and identified at the phylum level for samples.

In all samples, a different abundance of fungi sequences (95.1% in (A), 96.45% in (B), 99.68% in (C), and 99.58% in (D)) were registered. At the phylum level, the sequence abundance is presented in Figure 3. 

The number of sequences that were analyzed in our trimmed dataset for each sample demonstrated that three major fungal genus pathogens were found in leaf and soil samples in plants that were healthy and at stage I of infection, which were defined as *Ascochyta*, *Colletotrichum*, and *Fusarium*, as seen in Table 5. The occurrence of the basidiomycete Antrodia is remarkable.

Between samples represented by leaves from infected plants and soil collected around from infected plants, no common fungi were registered: only a different percentage of unclassified ones at the species level. 

Comparing soil samples, it can be observed that a higher abundance of different fungi characterizes the soil collected around infected plants (A) with *Ascochyta rabiei* being identified as the major pathogenic fungi species present. The same tendency may be observed in the plant samples with the mention that *Fusarium solani and Fusarium neocosmosporiellum* have a higher abundance in healthy plants (D). Moreover, the pathogenic fungi *Colletotrichum clidemiae* had been identified in the infected plant samples, and *Plectosphaerella* spp. had been identified in both infected and healthy plants.

The unique orders found in samples were represented by *Wallemiales* (0.47%), *Myrmecridiales* (0.4%), and *Magnaporthales* (0.28%) in infected plants (C) and *Capnodiales* (2.53%) in healthy plants (D).

### 3.3. Liquid Chromatography-Mass Spectrometry (LC-MS) Analysis

**Figure 4:**
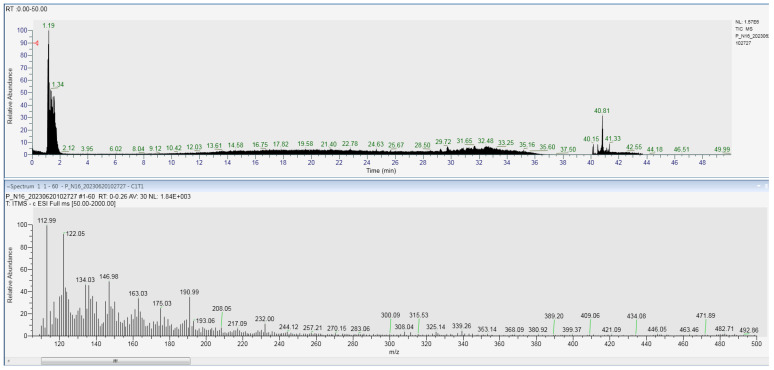
LC-MS chromatograms of compounds from *E. guinensis* samples.

The chromatograms were examined in full-scan mode, which revealed the presence of different compounds that were identified after the comparison with available standards, as listed in Table 6 and Figure 4. The compounds identified through the LC-MS method were represented by phenolic precursors (such as shikimic acid and caffeyl alcohol), flavonoids, epicatechin, kaempferol-7-O-neohesperidoside, naringenin-7-O-glucoside, isovitexin, rutin and flavonoid glycosides. 

## 4. Discussion

### 4.1. The Diagnosis and Recommendation Integrated System—DRIS

The estimation of the nutrients provides information concerning the different environmental pressures [47]. DRIS represents a technique applied in plant analysis (nutrient concentration) to diagnose the most limiting nutrient, exhibiting a weaker effect of environmental factors [48]. The DRIS methodology consists of the transformation of nutrients into indices (standardized by Gaussian distribution) and expressed reported to the degree of limitation from the greatest deficiency to highest excess. The nutrient index is expressed as positive and negative values. Positive indexes showed that the nutrients were in “excess” and negative indexes indicated that the nutrients were “deficient” in plants. 

Our DRIS analysis underlined a light deficiency of Ca^2+^ ions in healthy oil palms that increases during the infection stages of bud rot disease. It is known that calcium is an essential element in plants that serves as a constituent of cell walls and membranes, contributing to the structure of cells and upholding physical barriers against pathogens. By its structural role, plants deficient in calcium are more susceptible to pathogens, and exogenous calcium supply, in turn, has been shown to improve the plant’s resistance; calcium serves as a second messenger being interconnected with the signaling of other nutrients as well as pathogen attack [49]. So, the Ca deficiency registered in oil palm can be the main reason for the susceptibility of this crop to any pathogen attack and in our case could be the initial indication of bud rot in this crop. Mn is another deficient nutrient identified by DRIS analysis in oil palm, which is an important co-factor of different enzyme fundamentals for the biosynthesis of secondary metabolites associated with the shikimic acid pathway including phenolics, coumarins, lignin, and flavonoids [50]. Also, this deficiency can be one of the principal causes of the palm debility and susceptibility front of diseases. DRIS analysis revealed an excessive concentration of Fe in oil palm in all the disease stages and even in healthy palms. Iron is an essential nutrient for plants, playing an important role in the electron-transport chains of photosynthesis and respiration. At high levels, iron is toxic due to its capacity to act catalytically via the Fenton reaction to generate hydroxyl radicals, which can damage lipids, proteins, and DNA [51] and in conclusion can produce stress on oil palm levels that can make it more susceptible to pathogen damages. In the second stage of bud rot disease, oil palm presents a deficiency of Mg and Mn nutrients, not only Ca. 

Our results are based on those of Viégas et al., 2000 [52], which underline the influence of iron in the disease.

### 4.2. Metagenomic Analysis

Palm plantations have been affected by several diseases. The ones that stand out most among them are bud rot, root and trunk rot, lint disease, red ring, and others [53]. But rot is a disease with various effects from lower ones to the destruction of crops. There is a scale (CENIPALMA) for bud rot symptomatology: the healthy stage (with no lesions of the emerging leaf), stage 1 (lesions account for between 0.1% and 20% of the emerging leaf), stage 2 (the lesions cover between 40.1% and 60% of the emerging leaf) and stage 3 (lesions are spread between the 80.1% and 100% of the emerging leaf) [54]. 

Fungi, being decomposers, pathogens, and plant mutualists, have important roles in the ecosystem. These organisms are the most dominant groups in soil [55]. 

Although *Phytophthora palmivora* Butl has already been described as the causative agent for the first lesions, opportunistic pathogens might appear [56]: various fungi (*Fusarium* spp., *Colletotrichum* sp., *Thielaviopsis* sp., and *Rhizoctonia* sp., among others), bacteria (*Pseudomonas* sp. and *Erwinia* sp.) and insects (*Rhynchophorus palmarum*) that promote the rotting process. These biological agents have a high incidence in the death rate of the oil palm.

Previous studies carried out in Surinam [55] treating bud rot disease have isolated *Fusarium* spp., *Botrydiplodia* spp., *Colletotrichum* spp., and the *Erwinia amylovora* bacterium, while in Ecuador, a study regarding fungal diseases in oil palms identified *Fusarium* spp. followed by *Colletotrichum* spp., *Lasiodiplodia* spp., *Pestalotia* spp., *Nigrospora* spp., *Curvularia* spp. and *Trichoderma* spp. [57]. In the present study, an abundance of 17.41% of *Colletotrichum* spp. was found in the leaves of infected plants, while *F. solani* presented an abundance of 2.1% in infected plants and 11.54% in healthy plants in addition to the presence of *F. neocosmosporiellum* both in infected (1.99%) and healthy (10.43%) plants. 

Fungal diversity in oil palms plays a key role in the development of rot diseases, as described in the identification of several fungal pathogens, whilst in addition to the most abundant groups, *Colletotrichum* spp., an important plant pathogen, appears as the predominant group in diseased samples [58]. As the literature dictates, pathogens of the genus *Colletotrichum* are responsible for anthracnose diseases in various economically important crops [59], such as oil palms. Anthracnose, caused by *Colletotrichum* spp., is an important disease that affects palm trees especially since the fungus can survive in the debris of the previous year’s infected bunches for up to 8 months [60]. 

*Ascomycota* and *Basidiomicota* are the most common phylum found in all our samples. Our data are based on Wong et al., 2021 [61], which found that *Ascomycota* and *Basidiomicota* are the most frequent phylum found in the oil palm planted areas. *Ascomycota* was reported to be the dominant fungal group in plant tissues and different soil types and fertilizers [22]. It is predominantly terrestrial, generally, saprophytes on decaying plant debris contribute to the nutrient cycling in the ecosystems [62]. Also, *Basidiomycota* are important decomposers. 

Harvested oil palm trunks with their high moisture content are very susceptible to rot by wood decay via Basidiomycota. The brown rot fungus *Coniophora puteana* and the white rot species *Pleurotus ostreatus* decayed oil palm trunk samples with mass loss of up to 50% within 1 month of incubation [63].

Different steps of plant growth and development (like metabolism, nutrient regulation, reproduction, chlorophyll synthesis, carbohydrate production, fruit, and seed development, etc.) are performed by an adequate level of micro and macronutrients. The presence of decomposers is affecting the nutrients present in soils. A deficiency or excess of nutrients negatively influence the plant’s physiological, biochemical, and metabolic characteristics and can promote even abnormal growth and susceptibility in front of pathogens [64]. 

### 4.3. LC-MS Analysis

Recently, many bioactive compounds have been isolated that have contributed to the understanding of their role in the immune system of plants. Many natural compounds act as inducers of defense responses in plants [65]. The indirect action of bioactive compounds on plant cells stimulates the release of protein and lipid elicitors [66], leading to the synthesis of phytoalexins and pathogenesis-related proteins, the accumulation of callose and lignification of the cell wall, as well as increased activity of several defense enzymes, which protects plants against pathogens [67].

In the present study, different flavonoid compounds were identified through the LC-MS method. Flavonoids play an important role in the defense mechanism of the plant. In addition to acting as UV filters, signal molecules, allopathic chemicals, phytoalexins, detoxifying agents, and antimicrobials, flavonoids protect plants against biotic and abiotic stress [68]. The anti-pathogenic properties of flavonoids may result from their antioxidant properties influencing the deactivation of ROS generated by both pathogens and the plant as a result of infection [69].

Epicatechin is a known flavonoid with biological activity mainly attributed to its interaction with proteins and lipids and its antimicrobial properties essential for resistance in plant diseases [66], since it has been reported to inhibit appressorial melanization of the necrotrophic fungus *Colletotrichum kahawae* that causes coffee berry disease [70].

Rutin has also been identified in the plant sample, which is one of the huge families of flavonoids that was widely distributed in Plantae [71]. Like other flavonoids, rutin acts to reduce environmental stress, e.g., via UV-B screening, antioxidant activity, and disease resistance, through an increase in quercetin and rutinose concentrations [72].

Kaempherol and its glycosides, such as kaempferol 7-neohesperidoside identified in stage I of infection, have antibacterial, antiviral, antifungal, and antiprotozoal activities, as it has been reported in numerous papers [73,74,75]. Naringenin is a flavonoid classified as a flavanone, and it is widely distributed in several fruits and found in its glycoside form as well [76]. Recently, An et al., 2021 [77] found that naringenin induces pathogen resistance, suggesting that pathogen-accumulated naringenin leads to pathogen resistance, which is related to its known biological functions like other flavonoids. 

Flavones, a subclassification of flavonoids, had also been identified in the present study, such as isoorientin and isovitexin, which are described as C-glycosyl flavonoids and are found in different plants [78]. Flavones have a variety of functions for the plant defense mechanism, similar to flavonoids, including interactions between species like resistance to pathogens, symbiosis, protection against herbivory, and allelopathy [79,80].

## 5. Conclusions

The present study has focused on the biotic and abiotic factors represented by nutrient deficiency or excess, which trigger an increased susceptibility of the oil palm to any pathogenic attack and can cause a high disease incidence. The involvement of elements such as Ca, Fe, Mn, and Mg in the palm debility front of pathogens causing bud rot disease was proved. In the case of biotic factors, our study indicates that cumulative fungi are implied in bud rot disease. A very important role was played by the combination of biotic and abiotic factors. Numerous compounds (with protective roles) were identified in the leaves of symptomatic plants from stage I of the infection. The early identification of the determining factors of the disease (lack of minerals or pathogens present in the soil and/or plant) may contribute to the reduction in the disease incidence by isolating the affected specimens.

## Figures and Tables

**Table 1:** The Potential Response to the Application of Nutrients (RAPN) categories.

Potential Response to the Application of Nutrients (RPAN)	Inferior Limit (*α*)	Superior Limit
Cryodeficiency	−∞	−$\frac{4}{3}\sigma$
Prone to Deficiency	−$\frac{4}{3}\sigma$	−$\frac{2}{3}\sigma$
Nutritional Balance	−$\frac{2}{3}\sigma$	$\frac{2}{3}\sigma$
Prone to Excess or Toxicity	$\frac{2}{3}\sigma$	$\frac{4}{3}\sigma$
Excess or Toxicity	$\frac{4}{3}\sigma$	∞

**Table 2:** The ITS1 primer sequences were used for the analysis of the sample.

Primer Sequence for ITS1 Region	
Forward	ITS_fwd_1 CTTGGTCATTTAGAGGAAGTAA
	ITS_fwd_2 CTCGGTCATTTAGAGGAAGTAA
	ITS_fwd_3 CTTGGTCATTTAGAGGAACTAA
	ITS_fwd_4 CCCGGTCATTTAGAGGAAGTAA
	ITS_fwd_5 CTAGGCTATTTAGAGGAAGTAA
Reverse	ITS_rev_1 GCTGCGTTCTTCATCGATGC
	ITS_rev_2 GCTGCGTTCTTCATCGATGG
	ITS_rev_3 GCTACGTTCTTCATCGATGC
	ITS_rev_4 GCTGCGTTCTTCATCGATGT
	ITS_rev_5 ACTGTGTTCTTCATCGATGT

**Table 3:** Nextera adapter sequences.

Overhang Adapter Sequences	
Forward	TCGTCGGCAGCGTCAGATGTGTATAAGAGACAG-CTTGGTCATTTAGAGGAAGTAA
Reverse	GTCTCGTGGGCTCGGAGATGTGTATAAGAGACAG-GCTGCGTTCTTCATCGATGC

Adapted from Hoggard et al., 2018 [38].

**Table 4:** DRIS indexes and Response to Nutrient Application Potential (RPANs) for oil palm culture (IN, IK, IP, ICa, IMg, IS, IB, IFe, IMn, IZn, and ICu) in palms affected by the bud rot disease.

DRIS Index/IBN	Bud Rot Disease Infection Stages in Oil Palm							
	Healthy Plants		Stage I		Stage II		Stage III	
	Indices	RPANs	Indices	RPANs	Indices	RPANs	Indices	RPANs
IN	−396.74	−0.0	−411.27	−0.02	−517.24	−0.03	−500.30	−0.06
IP	−622.36	−0.44	−468.81	−0.15	−513.36	−0.02	−567.44	−0.18
IK	347.43	0.14	571.37	0.35	1012.20	0.70	774.99	0.50
Ica	−266.58	−0.41	−274.21	−0.39	−360.95	−0.33	−246.98	−0.65
IMg	−343.01	−0.15	−327.64	−0.21	−345.97	−0.37	−411.53	−0.14
IS	−414.41	−0.04	−350.33	−0.14	−391.10	−0.25	−430.52	−0.09
IB	175.26	0.82	204.91	0.68	271.70	0.61	295.41	0.47
ICu	451.92	0.12	367.41	0.09	349.42	0.36	432.07	0.09
IZn	513.27	0.25	442.28	−0.26	496.37	0.01	380.87	0.22
IMn	−365.38	−0.09	−524.50	0.65	−872.40	−0.56	−622.09	−0.28
Ife	865.11	0.77	773.28		921.22	0.61	897.74	0.64
IBN	432.86		428.73		550.18		505.45	
SD	490.50		476.06		633.27		565.73	

Legend: RAPNs—Response to Nutrient Application Potential, IBN—the nutritional balance index.

**Table 5:** The abundance (%) of sequences and identified at the species level for samples.

Category	Abundance %			
	A Infected Soil	B Healthy Soil	C Infected Plant	D Healthy Plant
Unclassified at the species level	25.8	30.89	31.03	19.78
*Ascochyta rabiei* (A)	20.79	9.3		
*Talaromyces ruber* (A)	9.76	7.71		
*Cryptococcus neoformans* (B)	6.58	5.5		
*Antrodia* sp. (B)	3.43	3.25		
*Saccharomyces* sp. (A)	3.37	7.9		
*Candida sake* (A)	3.06			
*Pyrenochaetopsis leptospora* (A)	1.98			
*Wallemia sebi* (B)		2.37		
*Acanthocorticium brueggemannii* (B)		1.99		
unidentified Hypocreales fam *Incertae sedis* sp.			22.91	5.47
*Colletotrichum clidemiae* (A)			17.41	
*Plectosphaerella cucumerina* (A)			12.93	2.33
*Acremonium stromaticum* (A)			5.49	
*Fusarium solani* (A)			2.1	11.54
*Fusarium neocosmosporiellum* (A)			1.99	1043
*Plectosphaerella oratosquillae* (A)			0.8	
*Cryptococcus nanyangensis* (B)				7.57
*Xenoacremonium recifei* (A)				4.21
*Fusarium ramigenum* (A)				3.88
*Wallemiales* (B)			0.74	
*Myrmecridiales* (A)			0.4	
*Magnaporthales* (A)			0.28	
*Capnodiales* (A)				2.53

Legend: A—Ascomycota, B—Basidiomycota.

**Table 6:** Total ion chromatogram (TIC) of the phenolic compounds identified in *E. guinensis* by LC-MS method in negative mode.

Identified Compound	Molecular Formula	Ion Adduct	Molecular Weight (g/mol)	LC-MS		
				[M-H]−	rt	Fr
Shikimic acid	C_7_H_10_O_5_	M − H	174.15	173.045	1.191	
Epicatechin	C_15_H_14_O_6_	M − H	290.07904	289.072	1.609	
(10E,15E)-9,12,13-trihydroxyoctadeca-10,15-dienoic acid	C_18_H_32_O_5_	M − H	328.4	327.218	21.467	3
[(4E)-7-acetyloxy-6-hydroxy-2-methyl-10-oxo-2,3,6,7,8,9-hexahydrooxecin-3-yl] (E)-but-2-enoate	C_16_H_22_O_7_	M + H	326.34	325.129	31.257	
[5-acetyloxy-3-(hydroxymethyl)-2-oxo-6-propan-2-ylcyclohex-3-en-1-yl] 3-methyl pentanoate	C_18_H_28_O_6_	M + H	340.4	339.181	32.56	
1-[2-methyl-6-[(2S,3R,4S,5S,6R)-3,4,5-trihydroxy-6-(hydroxymethyl)oxan-2-yl]oxyphenyl]ethanone	C_14_H_18_O_7_	M + H	298.29	311.114	32.046	21, 2, 12, 17, 5, 2
8-hydroxy-2,7,7,11,15-pentamethyl-5,12,16-trioxapentacyclo[9.8.0.0(2),.0,.0(1)(3),(1)]nonadec-13(18)-ene-3,17-dione	C_21_H_28_O_6_	M − H	376.4	377.102	1.14	
alpha, alpha-Trehalose	C_12_H_22_O_11_	M − H	342.297	387.115	1.14	15
Carnosine	C_9_H_14_N_4_O_3_	M + H	226.23	225.099	41.607	2, 3
Cystine	C_6_H_12_N_2_O_4_S_2_	M + H	240.3	239.017	41.913	9, 1
DOCOSANOL	C_22_H_46_O	M + H	326.6	325.348	34.004	
Ethylenediaminetetraacetic acid	C_10_H_16_N_2_O_8_	M − H	292.24	291.084	31.676	3
IS_N-BENZOYL-D5-GLYCINE	C_9_H_9_NO_3_	M − H	184.2	183.082	1.191	4
Canrenone	C_22_H_28_O_3_	M − H	340.2038	339.197	28.852	7, 3
Isoorientin	C_21_H_20_O_11_	M − H	448.38	447.093	13.429	3, 3
Massbank:IA000081 9-HODE	C_18_H_32_O_3_	M − H1	296.235	295.228	32.232	7
Massbank:IA000367 9-HOTrE	C_18_H_30_O_3_	M − H1	294.219	293.212	30.656	3
Citric acid	C_6_H_8_O_7_	M + H	192.12	191.02	1.191	2
n-Capric acid	C_10_H_20_O_2_	M − H	172.146	171	1.123	
Furosemide	C_12_H_11_ClN_2_O_5_S	M − H	330.0077	329	30.21	2
2-Hydroxyhippuric acid|2-hydroxyhippurate	C_9_H_9_NO_4_	M − H	195.05316	194.1	28.272	
Kaempferol-7-O-neohesperidoside	C_27_H_30_O_15_	M − H	594.15847	593.151	1.566	2
Naringenin-7-O-glucoside	C_21_H_22_O_10_	M − H	434.397	433.114	32.002	
Isovitexin	C_21_H_20_O_10_	M − H	432.381	431.098	1.744	1
Caffeyl alcohol	C_9_H_10_O_3_	M − H	166.176	165.056	1.158	
Massbank:PR309095 FA 18:2 + 2O	C_18_H_32_O_4_	M − H	312.45	311.222	29.94	
Massbank:PR309165 MGMG 18:3	C_27_H_46_O_9_	M + HCOO	514.656	559.312	31.229	3
Massbank:PR309165 MGMG 18:3	C_27_H_46_O_9_	M + HCOO	514.656	559.312	31.658	1
Massbank:PR309171 DGMG 18:3	C_33_H_56_O_14_	M + HCOO	676.8	721.363	29.236	3, 6
Coumaroyl + C6H9O8 (isomer of 844, 845, 846)	C_15_H_16_O_10_	M − H	356.28	355.065	1.557	8
Massbank:UT000256 9-HPODE	C_18_H_32_O_4_	M − H	312.23006	311.223	29.191	5
Massbank:UT000264 9-HpOTrE	C_18_H_30_O_4_	M − H	310.21441	309.207	28.062	1
Dodecylbenzenesulfonic acid	C_18_H_30_O_3_S	M-H	326.19157	325.184	29.94	8
Rutin	C_27_H_30_O_16_	M − H	610.15338	609.146	1.557	
Sesamin	C_20_H_18_O_6_	M + H	354.4	353.103	33.906	14
Sucrose	C_12_H_22_O_11_	M − H	342.3	341.109	1.14	
Thymol-beta-D-glucoside	C_16_H_24_O_6_	M + H	312.36	311.15	34.672	
Trihydroxy flavone-C-hexoside-C-pentoside	C_27_H_30_O_15_	M − H	594.5	563.141	1.557	2

## Data Availability

Not applicable.
